# Post-Salinity Stress Recovery of Black Calla Lily (*Arum palaestinum* Boiss.) Mediated by Plant Growth Regulators

**DOI:** 10.3390/plants15152262

**Published:** 2026-07-24

**Authors:** Mohamed A. Shahba, Mabruka Abubaira, Mohamed A. Darwish, Nehad F. Elshayeb

**Affiliations:** 1 Natural Resources Department, Faculty of African Postgraduate Studies, Cairo University, Giza 12613, Egypt; nehadelshayb@cu.edu.eg; 2 Biology Division, Front Range Community College, Larimer Campus, Fort Collins, CO 80526, USA; mabruka.abubaira@frontrange.edu; 3 National Research Centre, Fertilization Technology Department, Agricultural and Biological Research Institute, Giza 12622, Egypt; madarwishnrc@yahoo.com

**Keywords:** *Arum palaestinum*, stress recovery, abscisic acid, salicylic acid, ethephon, fusicoccin, ionic homeostasis, carbohydrate metabolism

## Abstract

Salinity stress severely limits growth, physiological function, and metabolic balance in the black calla lily (*Arum palaestinum* Boiss.), a medicinal and ornamental species native to Mediterranean regions. This study evaluated the effectiveness of selected plant growth regulators (PGRs) in enhancing black calla lily recovery following salinity stress. Seedlings were exposed to severe salinity (25 dS m^−1^) and subsequently treated with abscisic acid (ABA; 1.0–2.0 mM), salicylic acid (SA; 1.0–2.0 mM), fusicoccin (FC; 0.01–0.03 mM), or ethephon (E; 5–20 mM). Recovery was assessed using morphological, physiological, and biochemical indicators, including leaf color, leaf area, chlorophyll content, plant quality, K^+^/Na^+^ ratio, total nonstructural carbohydrates, reducing sugars, and proline content. All PGRs significantly improved recovery relative to untreated salt-stressed plants, with clear differences in efficacy. ABA was the most effective regulator, followed by ethephon, while SA and FC showed moderate benefits. Optimal ABA treatment (2.0 mM) resulted in near-complete recovery, characterized by improved leaf greenness and expansion, enhanced ionic homeostasis, increased carbohydrate reserves, and reduced accumulation of stress-related metabolites, particularly proline and reducing sugars, indicating alleviation of salinity stress and restoration of normal metabolic activity. These findings demonstrate that post-salinity recovery in *A. palaestinum* is strongly hormone-dependent and highlight the potential of ABA- and ethylene-based strategies to enhance salinity resilience.

## 1. Introduction

Saline environments affect plant growth in different ways including reduction in water uptake, gradual accumulation of ions to toxic levels, and reduction in nutrient accessibility [1]. The detrimental effects of salinity on plant growth include osmotic stress, ion toxicity, nutritional disturbances [2], damage to photosynthetic systems by excessive energy [3], and structural disorganization [4]. Plants respond to salinity stress through a number of physiological changes including lowered leaf osmotic potential and/or a loss of turgor potential, which can cause growth suppression. Salt tolerant plants often mediate stress by osmotic adjustment, therefore minimizing changes in turgor potential that affect plant growth responses linked to carbon dioxide assimilation and cell elongation [5]. Salt tolerance seems to depend mainly on the salt-induced accumulation of ions (Na^+^, Cl^−^, and Ca^2+^) and the shoot biosynthesis of organic osmolytes, both contributing to osmotic adjustment under stress. Active transport of these ions to the aerial part of the plants and high concentrations of glycine betaine have also been detected in non-stressed plants, indicating that these defense mechanisms against stress are at least partially constitutive [6].

Some growth regulators are known to help in stress tolerance. Many of the plant responses to stress occur via chemical signals such as the phytohormone abscisic acid (ABA) [7]. The endogenous concentrations of ABA in vegetative plant tissues rise in response to stresses that cause a plant water deficit. The protective effect of ABA is due to the promotion of stomatal closure to minimize water loss through transpiration, and then it mitigates stress damage through the activation of many stress-responsive genes, which collectively increase the plant stress tolerance. Refs. [8,9] concluded that spraying stressed wheat plants with exogenous plant growth regulators can effectively improve photosynthesis in fag leaves, enhance protective endoenzyme activity, reduce damage to cell membranes caused by the accumulation of MDA, and enhance the filling rate at the early filling stage, thereby stimulating grain formation, kernel number, and 1000-kernel weight. Recovery was most pronounced with 6-BA and SA. Didi et al. [10] provide evidence that 100 mg/L, 150 mg/L, and 200 mg/L of IAA, ABA, and GA3 treatments are effective for enhancing osmotic regulatory substances in *Nitraria tangutorum*, which offers an effective strategy for increasing tolerance to abiotic stress and improving the adaptability to harsh environments.

Plant species and cultivars within a species vary in their stress tolerance. These variations are due to variations in genes related to stress tolerance mechanisms and their interaction with the environments [11]. Stress-inducible genes can be functional or regulatory. Functional genes control water channels and other transporters, detoxification enzymes, protection molecules such as late embryogenesis abundant (LEA) proteins, key enzymes for osmolyte biosynthesis, or different proteases. Regulatory genes regulate the expression of the functional genes and include, among others, transcription factors, protein kinases and phosphatases, and those involved in abscisic acid biosynthesis [12].

The black calla lily (*Arum palaestinum* Boiss.) is one of approximately 26 species of the genus *Arum* (family *Araceae*), a group of tuberous perennial plants widely distributed across the Mediterranean basin. The plant typically grows 20–60 cm tall and is characterized by heart- to arrow-shaped leaves with a pungent, acrid taste, and an erect spadix enclosed by a pale green spathe marked with purplish spots [13]. *A. palaestinum* is native to North Africa, Europe, and Western Asia, with a notable presence in Palestine, Jordan, Lebanon, and Syria. Palestinian arum is called locally “Al-luf”. It is traditionally used as food, medicine, and an ornamental species. Its leaves are considered edible only after processing methods such as boiling, roasting, sun drying, or soaking in saline water to eliminate toxicity and are often cooked with olive oil [14,15]. In traditional medicine, *A. palaestinum* has been used to manage cancer, diabetes, obesity, cardiovascular disorders, food poisoning, and atherosclerosis, with several studies supporting its pharmacological potential [13,16].

The balance between carbohydrate production and consumption impacts salinity stress tolerance [17]. The decline in salinity tolerance in some species can be associated with reduced carbohydrate availability and reduced effectiveness of Na^+^ exclusion and K^+^ active uptake and transport [18,19]. Accordingly, fluctuations in these osmoregulators are expected during the recovery from stress.

Proline accumulates in larger amounts than other amino acids in salt-stressed plants [17]. Proline accumulation is the first response of plants exposed to salt stress and water-deficit stress and is thought to reduce injury to cells [20]. Proline may act as a signaling/regulatory molecule able to activate multiple responses that participate in the adaptation process to elevated salinity levels. Rapid accumulation of proline in tissues of many plant species in response to salt, drought, or temperature stress has been attributed to enzyme stabilization and/or osmoregulation [17,21]. However, other reports have indicated a negative effect of proline on salinity tolerance [22,23]. Accordingly, proper testing is required before making any conclusion regarding proline role in salinity tolerance in a given species, while fluctuations in its content are expected during the recovery from stress.

The literature has demonstrated that PGRs enhance plant germination, establishment, growth, and development. Research on the use of PGRs to improve vegetation restoration and remediation is, nevertheless, scarce. Based on current basic and applied research on PGRs, the agronomic products on the market may be useful for boosting growth and lowering stress effects. Fundamental and practical studies on PGR application rates, procedures, and results on individual species are needed [24]. Although many studies have investigated plant responses to stress through internal chemical signaling and adjustments in growth regulators, further research is needed to expand the evidence base supporting regulatory approval of plant growth regulator (PGR) products, as their use in stress recovery is relatively new, and only a limited number of studies have examined the effects of exogenous growth regulator applications during plant recovery from stress. The objectives of this study, therefore, are to (1) determine whether applications of abscisic acid (ABA), salicylic acid (SA), fusicoccin (FC), and ethephon (E) could promote *Arum palaestinum* Boiss recovery from salinity stress and growth; (2) determine the most effective concentrations of each growth regulator in the recovery of the stressed plants; (3) determine which evaluation criteria are associated with superior recovery rate; and (4) build the body of evidence supporting the regulatory approval for growth regulators’ use in agricultural operations to mitigate the symptoms of salt stress.

## 2. Results

**Figure 1:**
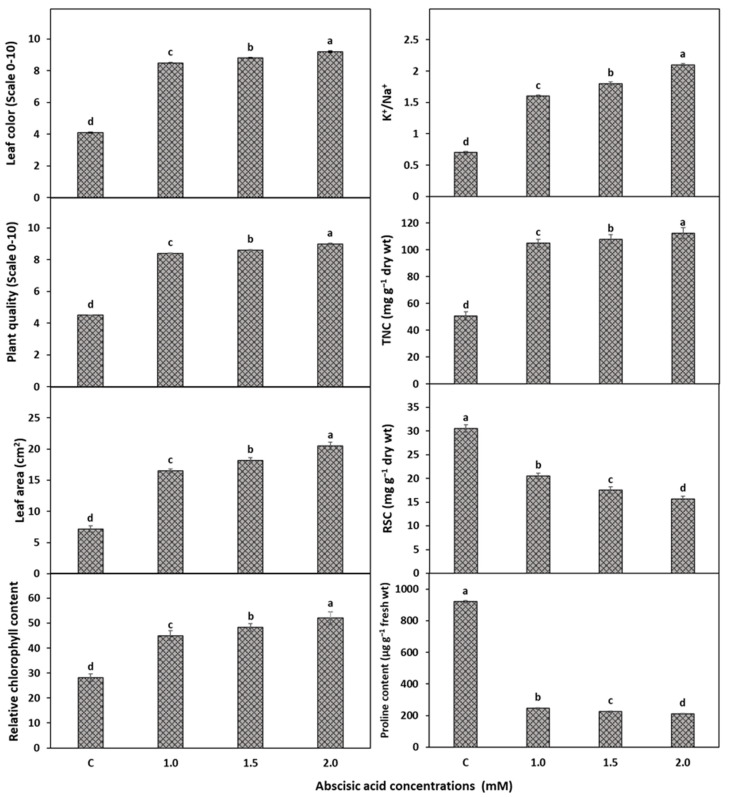
Effects of different concentrations of abscisic acid on growth, physiological, and biochemical traits of *Arum palaestinum* during recovery from salinity stress. Columns with different letters indicate significant differences at *p* ≤ 0.05. Error bars represent the standard error of the mean (SE).

**Figure 2:**
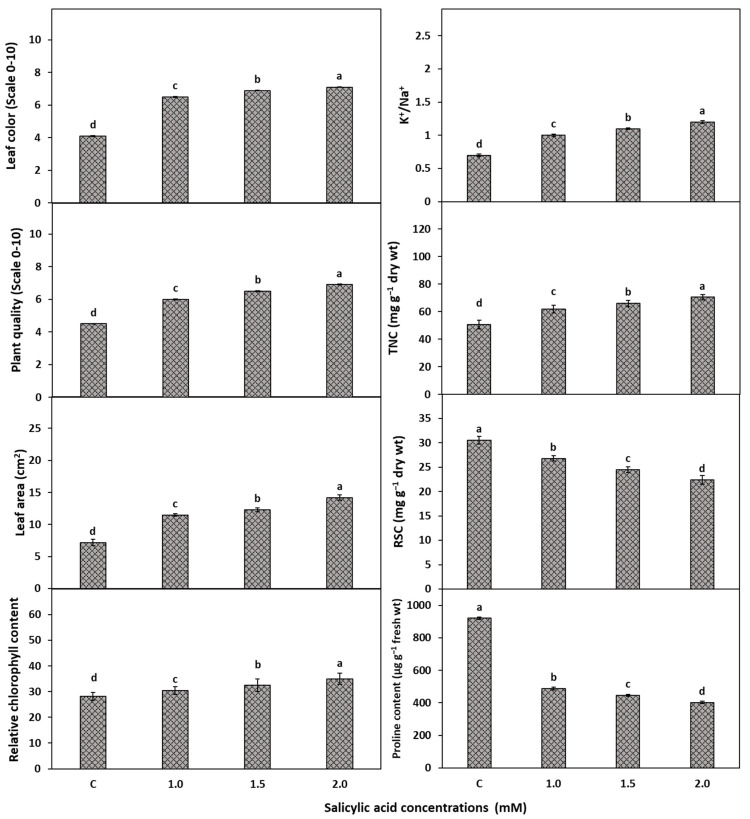
Effects of different concentrations of salicylic acid on growth, physiological, and biochemical traits of *Arum palaestinum* during recovery from salinity stress. Columns with different letters indicate significant differences at *p* ≤ 0.05. Error bars represent the standard error of the mean (SE).

**Figure 3:**
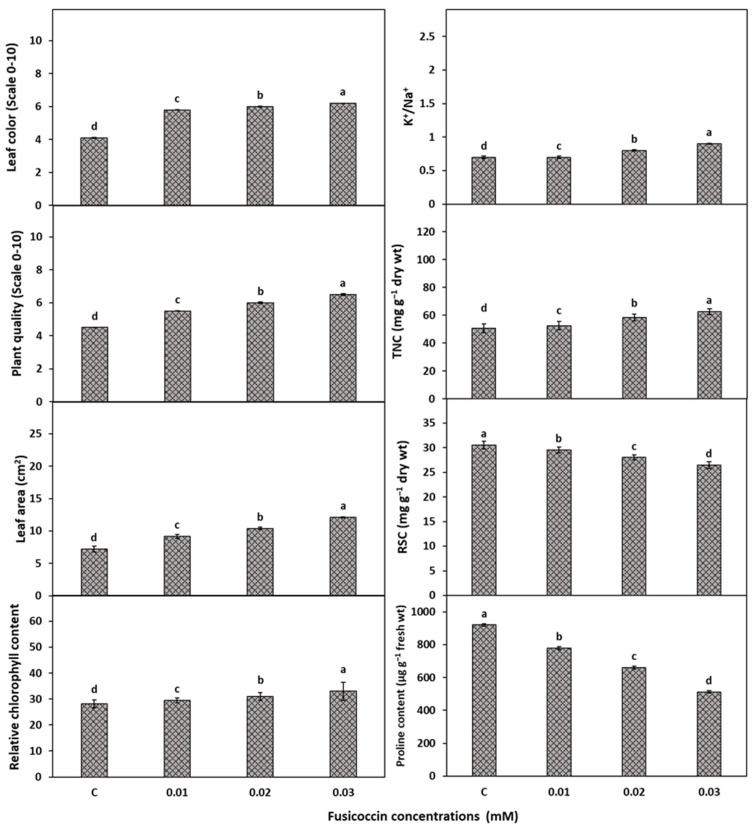
Effects of different concentrations of fusicoccin on growth, physiological, and biochemical traits of *Arum palaestinum* during recovery from salinity stress. Columns with different letters indicate significant differences at *p* ≤ 0.05. Error bars represent the standard error of the mean (SE).

**Figure 4:**
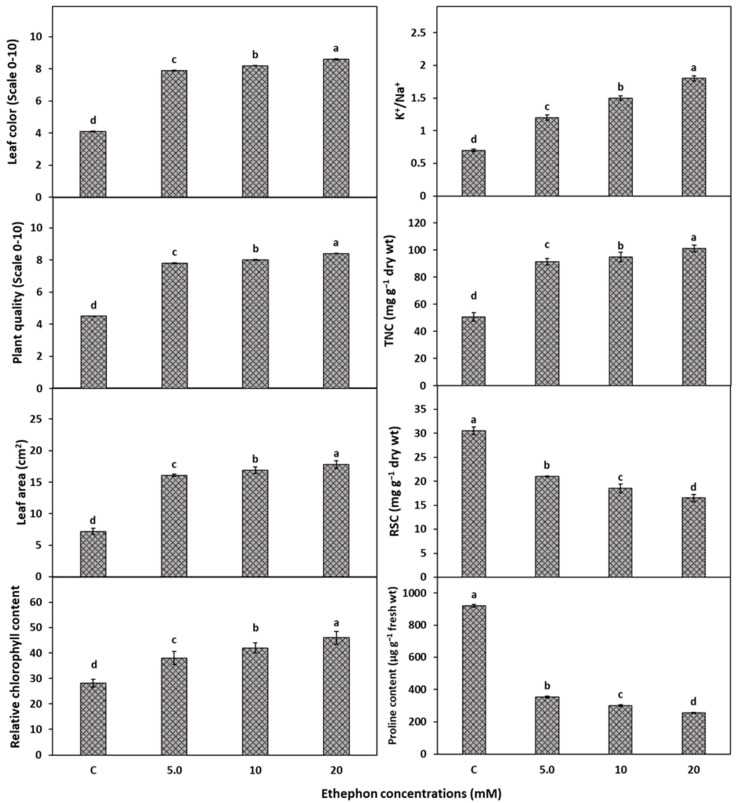
Effects of different concentrations of ethephon on growth, physiological, and biochemical traits of *Arum palaestinum* during recovery from salinity stress. Columns with different letters indicate significant differences at *p* ≤ 0.05. Error bars represent the standard error of the mean (SE).

**Figure 5:**
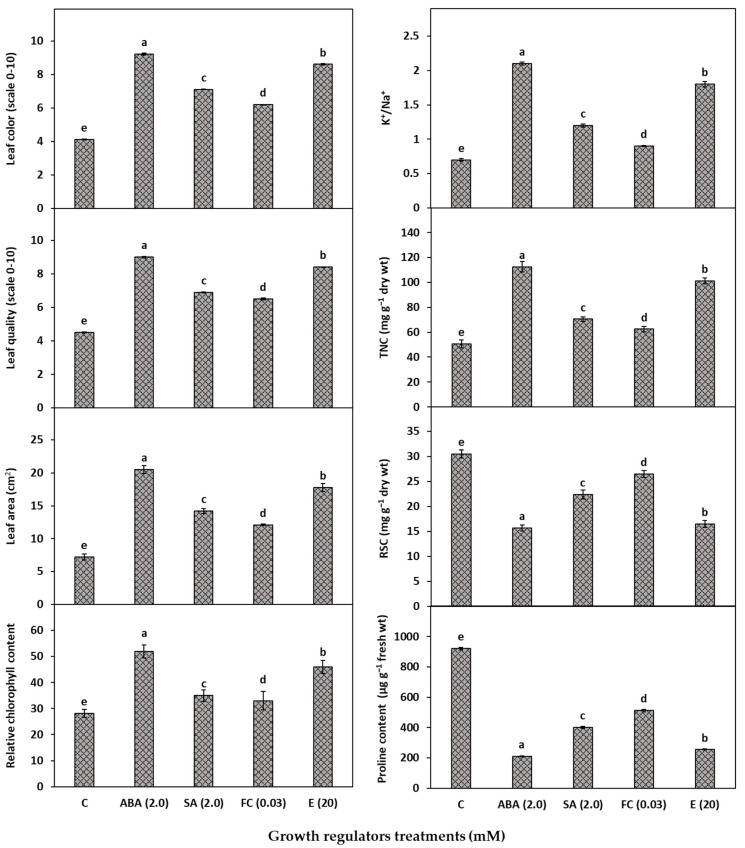
Effects of abscisic acid (ABA), salicylic acid (SA), fusicoccin (FC), and ethephon (E) at their optimal concentrations on the growth, physiological, and biochemical traits of *Arum palaestinum* during recovery from salinity stress. Columns with different letters indicate significant differences at *p* ≤ 0.05. Error bars represent the standard error of the mean (SE).

Salinity stress markedly impaired the growth, physiological performance, and metabolic balance of *Arum palaestinum* Boiss., as evidenced by chlorosis, reduced leaf expansion, disrupted ionic homeostasis, depletion of total nonstructural carbohydrate (TNC) reserves, and increased accumulation of stress-related metabolites, particularly reducing sugars and proline. The application of plant growth regulators significantly enhanced post-salinity recovery, although the extent of recovery varied among regulators and concentrations, as indicated by the analysis of variance (Table 1). Among the treatments, abscisic acid (ABA) consistently produced the greatest recovery (Figure 1), followed by ethephon (Figure 2), salicylic acid (SA) (Figure 3), and fusicoccin (FC) (Figure 4), with the overall treatment comparison presented in Figure 5.

### 2.1. Leaf Characteristics

#### 2.1.1. Leaf Color

Leaf color differed significantly among growth regulator treatments, concentrations, and their interactions (Table 1). Compared with the salt-stressed control (score = 4.1), all treatments improved leaf greenness in a concentration-dependent manner. ABA produced the greatest improvement, increasing leaf color scores from 8.5 at 1.0 mM to 9.2 at 2.0 mM (Figure 1), followed by ethephon, which reached 8.6 at 20.0 mM (Figure 2). More moderate improvements were observed with SA (7.1 at 2.0 mM) and FC (6.2 at 0.03 mM) (Figure 3 and Figure 4). Comparison of the optimal concentrations showed that ABA (2.0 mM) resulted in significantly greener leaves than all other treatments, followed by ethephon (20.0 mM), SA (2.0 mM), and FC (0.03 mM) (Figure 5). Overall, ABA produced the most pronounced recovery of leaf greenness, increasing the color score by more than twofold compared with the salt-stressed control, whereas SA and FC exhibited comparatively limited effects.

#### 2.1.2. Leaf Area

Salinity stress markedly reduced leaf expansion, whereas the application of all plant growth regulators significantly increased leaf area during recovery. The greatest leaf area was recorded with ABA (20.5 cm^2^ at 2.0 mM) (Figure 1), followed by ethephon (17.8 cm^2^ at 20.0 mM) (Figure 2), SA (14.2 cm^2^ at 2.0 mM) (Figure 3), and FC (12.1 cm^2^ at 0.03 mM) (Figure 4). Overall, recovery of leaf expansion followed the order ABA > ethephon > SA > FC, with ABA and ethephon producing significantly greater improvements than SA and FC at their optimal concentrations (Figure 5). Increasing regulator concentrations generally enhanced leaf area, particularly under ABA and ethephon treatments, whereas SA and FC showed comparatively smaller responses.

### 2.2. Plant Quality

Plant quality scores differed significantly among treatments and showed patterns consistent with those observed for leaf color and leaf area (Table 1). All plant growth regulators improved plant quality compared with the salt-stressed control. The highest quality score was recorded for ABA-treated plants (9.0) at 2.0 mM (Figure 1), followed by ethephon (8.4) at 20.0 mM (Figure 2), SA (6.9) at 2.0 mM (Figure 3), and FC (6.5) at 0.03 mM (Figure 4). Overall, plant quality ranked in the order ABA > ethephon > SA > FC (Figure 5). ABA and ethephon exhibited clear concentration-dependent improvements, with increasing concentrations resulting in higher quality scores, whereas SA and FC produced comparatively smaller responses.

### 2.3. Relative Chlorophyll Content

Relative chlorophyll content was significantly increased by all plant growth regulator treatments compared with the salt-stressed control (Table 1). ABA produced the highest chlorophyll content, reaching 52.0 SPAD units at 2.0 mM (Figure 1), followed by ethephon (46.0 SPAD units) at 20.0 mM (Figure 2), SA (35.0 SPAD units) at 2.0 mM (Figure 3), and FC (33.0 SPAD units) at 0.03 mM (Figure 4). Overall, chlorophyll recovery followed the order ABA > ethephon > SA > FC (Figure 5). The response was concentration-dependent, particularly for ABA and ethephon, which showed progressive increases with increasing concentrations, whereas SA and FC exhibited comparatively smaller improvements. At their optimal concentrations, ABA resulted in significantly higher chlorophyll content than the other treatments.

### 2.4. Ionic Homeostasis (K^+^/Na^+^ Ratio)

The application of all plant growth regulators significantly increased the K^+^/Na^+^ ratio compared with the salt-stressed control (Table 1). The highest K^+^/Na^+^ ratio was recorded under ABA treatment (2.1) at 2.0 mM (Figure 1), followed by ethephon (1.8) at 20.0 mM (Figure 2), SA (1.2) at 2.0 mM (Figure 3), and FC (0.9) at 0.03 mM (Figure 4). Overall, recovery of ionic balance followed the order ABA > ethephon > SA > FC (Figure 5). Increasing concentrations of ABA and ethephon resulted in progressive increases in the K^+^/Na^+^ ratio, whereas SA and FC showed comparatively smaller responses across concentrations.

### 2.5. Carbohydrate Metabolism

#### 2.5.1. Total Nonstructural Carbohydrates

Total nonstructural carbohydrate (TNC) content increased significantly following the application of all plant growth regulators compared with the salt-stressed control (Table 1). The highest TNC content was recorded under ABA treatment (112.5 mg g^−1^ DW) at 2.0 mM (Figure 1), followed by ethephon (101.2 mg g^−1^ DW) at 20.0 mM (Figure 2), SA (70.5 mg g^−1^ DW) at 2.0 mM (Figure 3), and FC (62.5 mg g^−1^ DW) at 0.03 mM (Figure 4). Overall, TNC accumulation followed the order ABA > ethephon > SA > FC (Figure 5). Increasing regulator concentrations generally resulted in progressive increases in TNC content, particularly under ABA and ethephon treatments.

#### 2.5.2. Reducing Sugars Content

Reducing sugar content declined significantly in all plant growth regulator treatments compared with the salt-stressed control (Table 1). The greatest reductions were observed under ABA treatment (15.7 mg g^−1^ DW) at 2.0 mM (Figure 1) and ethephon treatment (16.5 mg g^−1^ DW) at 20.0 mM (Figure 2). SA and FC treatments also reduced reducing sugar content but showed comparatively smaller effects (Figure 3 and Figure 4). The reduction in reducing sugar content followed the order ABA > ethephon > SA > FC at their optimal concentrations (Figure 5).

### 2.6. Proline Content

Proline content differed significantly among plant growth regulator treatments (Table 1). All treatments reduced proline accumulation compared with the salt-stressed control. The lowest proline content was recorded under ABA treatment (210.8 µg g^−1^ FW) at 2.0 mM (Figure 1), followed by ethephon at 20.0 mM (Figure 2), SA at 2.0 mM (Figure 3), and FC at 0.03 mM (Figure 4). The reduction in proline content followed the order ABA > ethephon > SA > FC (Figure 5). Higher regulator concentrations generally resulted in greater reductions in proline accumulation.

## 3. Discussion

### 3.1. Leaf Characteristics

#### 3.1.1. Leaf Color

Salinity stress substantially impaired growth and physiological performance in *A. palaestinum*, resulting in chlorosis, reduced leaf expansion, ionic imbalance, depletion of carbohydrate reserves, and accumulation of stress-associated metabolites. Similar responses to salinity have been reported in *A. palaestinum* under Mediterranean conditions [25]. The present study demonstrated that exogenous application of plant growth regulators enhanced post-salinity recovery, although the magnitude of improvement differed among treatments. ABA showed the greatest effectiveness in restoring plant performance, followed by ethephon, SA, and FC.

Recovery of leaf greenness represents an important indicator of chlorophyll stability and restoration of photosynthetic capacity after stress relief. In the present study, ABA produced the greatest improvement in leaf color, followed by ethephon, whereas SA and FC resulted in comparatively smaller increases. The superior response to ABA may be associated with its recognized role in coordinating recovery processes through regulation of oxidative balance, chloroplast protection, and stress-responsive metabolism. Previous studies have shown that ABA can enhance antioxidant defense systems and regulate chlorophyll degradation pathways, thereby reducing oxidative damage to chloroplast structures and promoting pigment retention under stress conditions [26,27]. The concentration-dependent improvement observed with ABA further supports its contribution to restoring physiological activity during the recovery phase.

Ethephon also substantially improved leaf greenness, likely through ethylene-mediated regulation of senescence and chloroplast maintenance. Ethylene has been reported to influence chlorophyll metabolism and delay senescence during stress recovery, contributing to improved pigment retention in crops exposed to adverse conditions [28]. The strong response observed under ethephon suggests that ethylene signaling can complement ABA-mediated recovery processes by supporting the restoration of photosynthetic tissues.

In comparison, SA and FC produced more moderate improvements in leaf color. The beneficial effects of SA may be related to its role in redox regulation and enhancement of antioxidant defenses, which can indirectly protect chlorophyll from oxidative degradation. Although FC is known to activate plasma membrane H^+^-ATPase and increase membrane energization, its comparatively limited effect on leaf color suggests that stimulation of membrane-related processes alone may not be sufficient to fully restore chlorophyll stability after severe salinity stress. Recovery of leaf pigmentation likely requires coordinated regulation of chloroplast protection, antioxidant activity, metabolic adjustment, and hormonal signaling rather than activation of a single physiological pathway.

#### 3.1.2. Leaf Area

Recovery of leaf expansion following salinity stress varied among the applied growth regulators, with ABA producing the greatest improvement, followed by ethephon, SA, and FC. These differences suggest that restoration of leaf growth requires coordinated regulation of several physiological processes, including water relations, carbon allocation, and growth-related signaling pathways, rather than simple resumption of cell expansion.

Although ABA is traditionally considered a growth-inhibitory hormone during active stress conditions, increasing evidence indicates that it also plays an important role during the recovery phase. ABA contributes to restoration of cellular hydration, regulation of stomatal function, maintenance of turgor, and redistribution of assimilates toward actively growing tissues, thereby supporting renewed leaf expansion after stress alleviation [29,30,31]. The concentration-dependent increase in leaf area observed under ABA treatment in the present study is consistent with these proposed recovery functions.

Ethephon also promoted substantial recovery of leaf expansion. The ethylene released from ethephon has been reported to regulate expansion activity and other cell wall-modifying processes involved in cell enlargement while also interacting with other hormonal pathways that coordinate growth resumption following stress relief [28]. These functions may explain the strong, although comparatively lower, stimulatory effect of ethephon relative to ABA.

The moderate response observed with SA may reflect its primary involvement in stress signaling and redox regulation rather than direct stimulation of leaf growth. Through enhancement of antioxidant capacity and reduction of oxidative damage, SA may indirectly support cellular activity and permit continued growth during recovery [32].

Fusicoccin produced the weakest improvement in leaf area and showed limited concentration-dependent effects. Although FC-mediated activation of plasma membrane H^+^-ATPase can enhance membrane energization and potentially support nutrient transport, these effects alone may not be sufficient to restore leaf expansion after severe salinity stress. Effective recovery of growth likely requires the integration of membrane function with hormonal regulation, water balance, carbon metabolism, and photosynthetic recovery. Similar studies have indicated that restoration of whole-plant growth after abiotic stress depends on coordinated metabolic reprogramming and recovery of photosynthetic and carbon-use processes rather than activation of a single physiological mechanism [2,4,6].

### 3.2. Plant Quality

Plant quality represents an integrated response of several morphological and physiological characteristics, including leaf greenness, leaf expansion, and overall plant vigor. Therefore, the higher quality observed following ABA treatment likely reflects coordinated improvement across multiple recovery processes rather than enhancement of a single trait.

The superior response to ABA is consistent with its recognized role as a central regulator of plant recovery from abiotic stress. ABA contributes to osmotic adjustment, maintenance of ion homeostasis, restoration of photosynthetic activity, and regulation of carbon metabolism, all of which are essential for improving plant performance after stress alleviation [33,34]. Recent studies further indicate that ABA signaling remains active during the recovery phase, facilitating the transition from stress defense toward renewed growth through continued regulation of gene expression, metabolic activity, and carbon allocation [31,35]. The concentration-dependent improvement in plant quality observed under ABA treatment in the present study supports its important role in coordinating recovery responses.

Ethephon also substantially improved overall plant quality, likely through ethylene-mediated regulation of leaf expansion, chlorophyll maintenance, and metabolic reactivation during recovery. Although the response was lower than that of ABA, ethylene has been reported to interact with other hormonal pathways and contribute to stress recovery by regulating growth-related processes and resource allocation [28]. The superior recovery of leaf growth observed with ABA and ethephon may also reflect coordinated interactions among multiple phytohormones rather than the action of individual regulators alone. Recent studies indicate that plant growth recovery following abiotic stress is governed by extensive hormonal crosstalk involving ABA, ethylene, auxin, and other signaling pathways that collectively regulate cell expansion, tissue development, and resource allocation during stress recovery [36]. Although these signaling interactions were not investigated in the present study, they provide a plausible framework for interpreting the differential growth responses observed among the tested plant growth regulators and warrant further molecular investigation.

In comparison, SA and FC resulted in more limited improvements in plant quality. The effects of SA may be associated mainly with enhancement of antioxidant capacity and maintenance of cellular redox balance, whereas FC primarily influences membrane energization and transport processes. These functions may provide partial support for recovery but may not encompass the broad physiological coordination required to fully restore plant vigor after severe salinity stress. The results indicate that regulators capable of integrating multiple physiological processes, particularly ABA and ethylene, are more effective in promoting whole-plant recovery following salinity stress.

### 3.3. Relative Chlorophyll Content

Recovery of chlorophyll content represents an important indicator of restoration of photosynthetic capacity following salinity stress. In the present study, all plant growth regulators improved relative chlorophyll content compared with salt-stressed plants, with ABA producing the greatest enhancement, followed by ethephon, while SA and FC showed comparatively moderate effects.

Salinity-induced chlorophyll loss is commonly associated with oxidative damage to chloroplast structures, disruption of thylakoid membranes, inhibition of chlorophyll biosynthesis, and accelerated pigment degradation [37,38]. Therefore, recovery of chlorophyll content reflects not only pigment restoration but also repair of the photosynthetic apparatus and reactivation of metabolic processes.

The superior effect of ABA on chlorophyll recovery is consistent with its established role in protecting chloroplast function during and after abiotic stress. Previous studies have shown that ABA enhances antioxidant enzyme activity, reduces reactive oxygen species accumulation, and preserves chloroplast ultrastructure, thereby limiting oxidative damage during stress recovery [26]. In addition, ABA regulates the expression of genes involved in chlorophyll biosynthesis and chloroplast maintenance, supporting sustained photosynthetic activity after stress alleviation [39]. Improved osmotic regulation and restoration of stomatal function under ABA treatment may further contribute to chlorophyll stability by reducing secondary oxidative stress.

Ethephon also substantially enhanced chlorophyll recovery, likely through ethylene-mediated regulation of chloroplast maintenance and pigment metabolism. Ethylene has been reported to delay chloroplast senescence and promote the expression of chlorophyll biosynthetic genes and chloroplast-associated proteins during recovery from salinity stress, which may explain the strong improvement observed following ethephon application [28].

In contrast, SA and FC produced smaller increases in chlorophyll content. The beneficial effects of SA are likely related to its role in antioxidant defense and redox regulation, which can reduce oxidative damage but may provide less direct stimulation of pigment restoration under severe salinity stress. Similarly, the limited response to FC suggests that activation of plasma membrane H^+^-ATPase and enhancement of membrane energization alone are insufficient to fully restore chloroplast function. Effective recovery of photosynthetic capacity requires coordinated regulation of pigment metabolism, antioxidant protection, carbon assimilation, and hormonal signaling pathways. Collectively, these findings highlight the dominant roles of ABA and ethylene in restoring chlorophyll stability and photosynthetic performance during post-salinity recovery.

### 3.4. Ionic Homeostasis (K^+^/Na^+^ Ratio)

Maintenance of a high K^+^/Na^+^ ratio is widely recognized as a key component of salinity tolerance because it reflects the ability of plants to restrict Na^+^ accumulation while maintaining adequate K^+^ levels required for enzyme activity and cellular metabolism. The marked improvement observed following ABA application is consistent with previous studies reporting that ABA contributes to ion homeostasis during recovery from salinity stress.

The pronounced improvement in the K^+^/Na^+^ ratio observed under ABA treatment is consistent with its reported role in regulating ion balance during salinity stress recovery. Previous studies suggest that ABA signaling may enhance Na^+^ exclusion through activation of the SOS (Salt Overly Sensitive) pathway, particularly SOS1-mediated Na^+^ efflux at the plasma membrane, while potentially modulating high-affinity K^+^ transporters (HKT1-type proteins) to limit Na^+^ translocation to shoots and maintain cytosolic K^+^ levels [40,41]. Recent studies further indicate that ABA may fine-tune ion transporter activity through ABA-responsive transcription factors and post-translational regulation, contributing to membrane potential stabilization and the maintenance of enzyme functionality under prolonged salt stress [27,35].

The substantial improvement in the K^+^/Na^+^ ratio following ethephon application is also consistent with previous reports describing the role of ethylene in ion homeostasis during stress recovery. Ethylene released from ethephon has been suggested to enhance root ion selectivity through modifications in root architecture, increased Na^+^ sequestration into vacuoles, and interaction with ABA-mediated signaling pathways. Furthermore, previous research indicates that ethylene may promote the expression or activity of vacuolar Na^+^/H^+^ antiporters (NHXs) and strengthen antioxidant defenses, thereby potentially contributing to improved ion balance under saline conditions [42]. Salicylic acid and fusicoccin resulted in more moderate improvements in ionic balance (Figure 5). Salicylic acid has been reported to influence membrane stability and redox signaling, which may indirectly affect ion transport; however, its effects on specific Na^+^ and K^+^ transport mechanisms appear to be more variable and dependent on species and stress conditions, as was previously reported [43]. Fusicoccin, which is primarily associated with plasma membrane H^+^-ATPase activation, may enhance nutrient transport under favorable conditions; however, its limited effect in the present study suggests that stimulation of membrane energization alone may not be sufficient to effectively restore Na^+^ exclusion mechanisms during recovery from severe salinity stress. The differential responses among treatments suggest that growth regulators influence ionic recovery through distinct and potentially complementary pathways. The superior performance of ABA and ethylene in restoring a favorable K^+^/Na^+^ ratio is consistent with previous evidence highlighting their important roles in regulating ion transport, compartmentalization, and potassium conservation during plant responses to salinity.

Although the present study evaluated whole-leaf physiological responses, the underlying regulation of ion homeostasis is likely to involve tissue- and cell-type-specific signaling networks that were not examined here. Recent advances in spatial transcriptomics indicate that hormonal regulation and ion transport during salinity responses are highly cell-type dependent, providing opportunities to better resolve the mechanisms underlying post-stress recovery in future studies [44].

### 3.5. Carbohydrate Metabolism

#### 3.5.1. Total Nonstructural Carbohydrates

Recovery from salinity stress was accompanied by a substantial increase in total nonstructural carbohydrate reserves, particularly following ABA and ethephon application. Restoration of TNC is generally considered an indicator of improved photosynthetic performance and recovery of carbon balance because carbohydrate reserves provide both energy and structural substrates required for renewed growth.

The superior performance of ABA is consistent with previous reports indicating that ABA regulates source–sink relationships, promotes starch biosynthesis, stabilizes sucrose metabolism, and modulates sugar transporter expression during stress recovery [30]. Recent studies further suggest that ABA coordinates carbohydrate metabolism through interactions with the SnRK1 and TOR signaling pathways, thereby optimizing carbon allocation and energy homeostasis during the transition from stress adaptation to renewed growth [44,45,46].

Ethephon also substantially increased TNC accumulation. Ethylene has been reported to maintain photosynthetic activity, delay senescence, and facilitate redistribution of assimilates toward actively growing tissues, thereby supporting restoration of carbohydrate reserves during post-stress recovery [47].

SA produced a more moderate increase in TNC, consistent with its primary role in protecting the photosynthetic apparatus through antioxidant activity rather than directly regulating long-term carbon storage. Likewise, FC resulted in comparatively limited carbohydrate accumulation. Although activation of plasma membrane H^+^-ATPase may enhance membrane transport and short-term assimilate movement, available evidence suggests that membrane energization alone has limited capacity to restore long-term carbon reserves without coordinated regulation of photosynthesis and hormonal metabolism [2,4,40,41]. FC’s weaker effect on TNC accumulation in the present study suggests that membrane activation alone may have limited capacity to enhance long-term carbon reserve restoration during post-salinity recovery. These findings indicate that successful recovery of carbohydrate reserves depends on integrated regulation of photosynthesis, carbon allocation, and hormonal signaling, with ABA and ethylene providing the greatest contribution under the conditions of the present study.

#### 3.5.2. Reducing Sugar Content

Reducing sugars accumulated under salinity stress primarily as compatible solutes contributing to osmotic adjustment. Their decline following the application of plant growth regulators therefore suggests reduced osmotic stress and renewed utilization of carbohydrates for growth and metabolism. The marked decline in reducing sugars following hormonal treatments indicates a metabolic shift from stress-induced osmoprotection to the active utilization of carbohydrates for respiration, cell wall biosynthesis, and growth-related processes, thereby signaling physiological recovery [48,49]. ABA-induced reductions in reducing sugars are consistent with its role in reprogramming carbon metabolism during stress alleviation. ABA promotes the conversion of soluble sugars into starch and structural carbohydrates while simultaneously enhancing mitochondrial respiration and energy production required for repair and growth. Recent studies have demonstrated that ABA coordinates sugar signaling with SnRK1–TOR regulatory networks, thereby facilitating the transition from survival metabolism to anabolic growth once stress intensity is reduced [35,44].

Ethephon produced a comparable response. Ethylene has been associated with increased sink activity, enhanced glycolytic metabolism, and redistribution of soluble sugars toward developing tissues during recovery, thereby reducing the requirement for osmotic sugar accumulation [50].

SA and FC produced comparatively smaller reductions in reducing sugars, suggesting partial alleviation of osmotic stress but less efficient restoration of carbon partitioning. Overall, the pronounced decline in reducing sugar content observed following ABA and ethephon application is consistent with recovery of metabolic activity and re-establishment of normal carbon utilization following salinity stress.

### 3.6. Proline Content

Proline is widely recognized as an important compatible solute that accumulates in plants exposed to salinity stress, contributing to osmotic adjustment, stabilization of proteins and membranes, and scavenging of reactive oxygen species. Consequently, elevated proline concentrations generally reflect continued stress, whereas declining proline levels during recovery are often associated with restoration of cellular homeostasis.

The marked reduction in proline observed following ABA application is consistent with previous reports suggesting that ABA participates in regulating proline metabolism during stress recovery. ABA has been reported to suppress the expression of proline biosynthetic genes, such as P5CS, while promoting proline degradation through increased proline dehydrogenase activity, thereby facilitating the redistribution of nitrogen and carbon toward growth-related metabolism [35]. Recent studies further suggest that ABA integrates proline metabolism with redox regulation and cellular energy signaling, supporting the transition from stress acclimation to renewed growth following stress alleviation [51,52].

Ethephon also substantially reduced proline accumulation. Ethylene has been associated with improved ionic and osmotic balance, enhanced antioxidant capacity, and reduced dependence on osmoprotective metabolites during recovery. Previous studies suggest that ethylene-mediated improvement of cellular homeostasis may decrease the requirement for continued proline synthesis as growth resumes [53].

SA and FC produced comparatively smaller reductions in proline content, indicating partial alleviation of stress but a less complete restoration of metabolic homeostasis. SA likely reduced proline indirectly through improved antioxidant protection and mitigation of oxidative stress, whereas the comparatively limited response observed with FC further suggests that activation of plasma membrane H^+^-ATPase alone is insufficient to fully reverse the metabolic adjustments associated with prolonged salinity stress. The decline in proline accumulation, particularly following ABA and ethephon application, is consistent with effective post-salinity recovery characterized by improved osmotic adjustment, restoration of ionic balance, and renewed growth-related metabolism.

### 3.7. Overall Physiological Implications, Limitations, and Future Research

Across the evaluated morphological, physiological, and biochemical parameters, abscisic acid (ABA, 2.0 mM) produced the most pronounced recovery response in *Arum palaestinum* Boiss. following salinity stress, followed by ethephon (20.0 mM), whereas salicylic acid (SA, 2.0 mM) and fusicoccin (FC, 0.03 mM) showed comparatively moderate effects (Figure 5). The superior performance of ABA and ethephon was associated with improved leaf greenness, leaf expansion, chlorophyll content, K^+^/Na^+^ balance, and total nonstructural carbohydrate (TNC) accumulation, together with reduced proline and reducing sugar levels. Collectively, these responses indicate a shift from stress-associated metabolic adjustment toward recovery processes supporting renewed growth and physiological function. The greater effectiveness of ABA is consistent with its established role in coordinating multiple physiological processes involved in salinity adaptation and recovery, including water relations, ion homeostasis, and metabolic regulation. Previous studies have demonstrated that ABA contributes to maintaining cellular stability under salinity by regulating osmotic adjustment, ion transport, and restoration of photosynthetic activity during stress recovery [26,40,41]. The positive effects of ethephon may be related to ethylene-mediated regulation of recovery-associated processes, as ethylene has been reported to interact with other hormonal pathways and influence stress signaling, antioxidant capacity, and metabolic adjustment under saline conditions [28,42,47]. The reduction in proline accumulation observed, particularly under ABA and ethephon treatments, may reflect reduced stress intensity and a transition toward growth-oriented metabolism, consistent with the reported role of hormonal regulation in controlling proline turnover and metabolic reprogramming during stress recovery [35,51,52].

Salicylic acid and fusicoccin also improved several recovery-related traits but were less effective than ABA and ethephon. The beneficial effects of SA may be associated with its role in redox regulation and stress signaling, which can contribute to improved cellular protection under adverse conditions [32,43]. In contrast, the relatively weaker response observed with FC suggests that activation of plasma membrane energization alone may not be sufficient to restore the complex physiological processes affected by salinity stress. Although H^+^-ATPase activation can contribute to membrane potential maintenance, nutrient transport, and cellular homeostasis, effective recovery requires coordinated regulation of ion balance, photosynthetic activity, carbon allocation, and hormonal signaling networks [2,4,41,42]. Therefore, the limited effects of FC on leaf expansion, chlorophyll recovery, and carbohydrate accumulation may indicate that membrane energization alone cannot fully compensate for the broader metabolic and physiological adjustments required during post-salinity recovery. Similar conclusions have been reported, indicating that recovery from abiotic stress depends on integrated metabolic reprogramming and restoration of photosynthetic and carbon-use processes rather than a single regulatory mechanism [6,48,49].

The differential responses among the tested growth regulators highlight their distinct but complementary roles in facilitating post-salinity recovery. Under the conditions of this study, ABA and ethephon were the most effective treatments for improving growth performance and physiological recovery of *A. palaestinum*. These findings provide a basis for further investigation of hormone-based approaches to enhance recovery capacity in salt-sensitive ornamental species. However, the present study focused primarily on above-ground responses; therefore, future studies should incorporate the tuberous rhizome of *A. palaestinum* to provide a more complete understanding of whole-plant recovery mechanisms. Analysis of below-ground tissues would help clarify tissue-specific ion distribution, carbohydrate storage dynamics, and the contribution of storage organs to Na^+^ and K^+^ regulation during recovery from salinity stress.

Furthermore, because the current experiment was conducted under controlled greenhouse conditions, environmental factors such as field-level salinity fluctuations, soil heterogeneity, and plant–soil interactions may influence the magnitude and consistency of growth regulator responses. Field-based validation will therefore be necessary to determine the practical applicability of these treatments. In addition, interactions among ABA, ethephon, SA, and FC were not examined in this study; however, hormonal crosstalk may result in synergistic or antagonistic responses depending on stress severity and developmental stage. Future integration of physiological analyses with transcriptomic, metabolomic, and spatial approaches will provide deeper insight into the regulatory networks controlling post-salinity recovery and may support the development of more effective strategies for improving resilience in salt-sensitive plant species.

## 4. Materials and Methods

### 4.1. Cultural Practices and Treatments

Experiments were conducted in the spring of 2023 and repeated in the spring of 2024. Seeds of *A. palaestinum* were collected from their growing habitats in the Bargash protected area, Irbid, Jordan, during the spring of 2015. The collected seeds were thoroughly air-dried after collection, sealed in moisture-proof containers, and stored under controlled laboratory conditions at 4 °C in a low-humidity environment to maintain their viability. Prior to the experiments, germination tests confirmed that the stored seeds retained sufficient viability for experimental use. Seedlings of *A. palaestinum* were germinated under laboratory conditions, where seeds were sown on sterile germination blotter papers lined in 9 cm diameter Petri dishes. In each dish, 50 seeds were placed on each germination blot. Germination blots were moistened with 20 mL of tap water. Petri dishes were sealed with parafilm and placed in a germinator at 15 °C. The germinator’s light source was six cool white fluorescent bulbs, which emit approximately 10.25 µmol m^−2^ s^−1^ of light. The germinator was set for 16 h of light and 8 h of dark. Germinated seedlings were transported to the greenhouse (daytime temperatures ranged from 25.0 to 30.0 °C, and nighttime temperatures ranged from 20.0 to 25.0 °C) and transplanted into 30 cm pots containing potting mix. Adjusted to be neutral, commercial potting soil (Scotts Metro-Mix 350, Scotts-Sierra Horticultural Products) was used. Greenhouse temperatures were set to match the optimal ranges for *A. palaestinum* in its original habitat (25 °C). Active radiation for efficient photosynthesis above the plants at 10 h was around 1000 µmol m^−2^ s^−1^. The plants were maintained under these conditions until complete recovery and establishment and continued growing for two weeks until seedlings had three leaves; then, they were transplanted into 15 cm diameter and 50 cm long PVC tubes (lysimeter columns) containing PRO-MIX^®^ potting mix with mycorrhizae and biofungicide (Premier Tech Horticulture, USA).

### 4.2. Inducing Salinity Stress

All columns were placed in the greenhouse in holding racks. In a previous study, Shahba et al. [25] indicated that *A. palaestinum* collected from the Mediterranean area could tolerate irrigation water with a salinity level up to 15 dS m^−1^ after they tested the effect of 5, 15, and 25 dS m^−1^ salinity treatments. Accordingly, control plants were irrigated throughout the experiment with tap water, while salinity-treated plants received irrigation water adjusted to an electrical conductivity (EC) of 25 dS m^−1^ using an Instant Ocean^®^ salt mixture. Salinity stress was considered established when plants consistently exhibited visible chlorosis, reduced leaf expansion, and loss of leaf turgor, accompanied by a measurable reduction in SPAD chlorophyll values relative to the control plants [54].

### 4.3. Experimental Design

The experiment was arranged as a randomized complete block design (RCBD) with four blocks. Each block contained 48 experimental units (PVC tubes), representing three tubes for each of the 16 treatment combinations (four growth regulators × four concentrations, including the untreated control). The three tubes assigned to each treatment within a block were averaged to obtain a block mean, and the four blocks were considered the independent experimental replicates for statistical analysis. Prior to treatment application, plants were selected to ensure uniformity in size, height, number of leaves, and severity of salinity stress. Four plant growth regulators were evaluated, each at four concentrations, including the untreated control. Treatments were applied weekly through the irrigation water. Abscisic acid (ABA) was applied at 0, 1.0, 1.5, and 2.0 mM; salicylic acid (SA) at 0, 1.0, 1.5, and 2.0 mM; fusicoccin (FC) at 0, 0.01, 0.02, and 0.03 mM; and ethephon (E) at 0, 5, 10, and 20 mM. The treatments were continued for two months. Evapotranspiration was monitored weekly using four representative tubes that served as lysimeters. These tubes were irrigated to container capacity, allowed to drain for 2 h, and then weighed. Each tube was subsequently reweighed at 24 h intervals, and the daily change in weight was used to estimate evapotranspiration.

### 4.4. Data Collection

During the experiment, leaf color, leaf area, and plant height were monitored weekly for all experimental plants. Individual plant height was measured from the soil surface to the highest point of the plant. Visual leaf color was rated weekly using a 0–10 scale (brown to green), with a rating of 6.0 or higher considered acceptable, indicating sufficient greenness to maintain normal physiological function. Leaf area was measured using a LI-COR leaf area meter (LI-3000 (LI-COR Biosciences, Lincoln, NE, USA)), and relative chlorophyll content was determined using a SPAD-502 Chlorophyll Meter (Konica Minolta, Osaka, Japan). For biochemical analyses, at the end of the experiment, three representative leaf subsamples were collected from each treatment for determination of proline, total nonstructural carbohydrates (TNC), reducing sugar content (RSC), and tissue K^+^ and Na^+^ concentrations.

Representative leaf samples were collected at the end of the experiment for determination of proline content (µg g^−1^ fresh weight), total nonstructural carbohydrates (TNC; mg g^−1^ dry weight), reducing sugar content (RSC; mg g^−1^ dry weight), and tissue K^+^ and Na^+^ concentrations. Samples were collected from representative plants within each treatment and block, and the resulting measurements were averaged according to the experimental design described above.

For carbohydrate analyses, twenty leaves with petioles representing both young and mature leaves were randomly collected, washed with deionized water, and prepared for analysis. Samples (5 g) were freeze-dried (Genesis 25 LL Lyophilizer, Virtis, Gardiner, NY, USA), ground using a Wiley mill, passed through a 425 µm sieve, and stored in airtight vials at −20 °C until analysis. Total nonstructural carbohydrate content was determined according to the method described by Chatterton et al. [55]. Briefly, 25 mg of freeze-dried tissue was incubated in 5 mL of 0.1% clarase solution at 38 °C for 24 h. Hydrochloric acid (0.5 mL; 50%, *v*/*v*) was then added, and the solution was incubated at room temperature for 18 h. The pH was adjusted to 5–7 using 10 and 1 N NaOH solutions. The resulting extract was used for TNC determination using a spectrophotometer at 515 nm (DU640 (Beckman Instruments, Fullerton, CA, USA)). Reducing sugar content was determined by extracting 25 mg of freeze-dried powder in 10 mL of 0.1 M phosphate buffer (pH 5.4) for 24 h at room temperature. An aliquot (0.2 mL) of the extract was used for RSC determination following the spectrophotometric procedure described for TNC analysis.

Proline content was determined according to the method of Bates et al. [56] as modified by Torello and Rice [57] using 0.5 g of fresh leaf tissue. Fresh samples were used to minimize potential degradation or losses associated with drying. Samples were immediately frozen in liquid nitrogen, ground, and homogenized in 10 mL of 3% sulfosalicylic acid solution. The extracts were shaken for 1 h and filtered using Whatman No. 2 filter paper. Two milliliters of filtrate were reacted with 2 mL of ninhydrin reagent (1.25 g ninhydrin dissolved in 30 mL glacial acetic acid and 20 mL of 6 M H_3_PO_4_) and 2 mL of glacial acetic acid. The reaction mixture was heated at 100 °C for 1 h in a water bath and then rapidly cooled in an ice bath, and the proline concentration was determined spectrophotometrically at 520 nm. Concentrations were calculated using a standard curve prepared with known proline concentrations.

For ion analysis, shoot tissues were washed with deionized water and dried at 70 °C until constant weight. Dried samples were ground using a Wiley mill and passed through a 425 µm sieve. One gram of dried tissue was ashed at 500 °C for 7 h, and the resulting ash was dissolved in 10 mL of 1 N HCl and diluted with deionized water. Sodium and potassium concentrations were determined using inductively coupled plasma atomic emission spectrophotometry (Model 975 Plasma AtomComp (Thermo Jarrell Ash Corporation, Franklin, MA, USA)).

### 4.5. Data Analysis

Initial analysis of variance indicated no significant differences between the two experimental runs; therefore, data from both experiments were pooled. The effects of growth regulator treatments, regulator concentrations, and their interactions were evaluated using ANOVA procedures in SAS/STAT version 15.1 [58]. The experimental unit was the block mean generated from the three tubes assigned to each treatment within each block, resulting in four independent replicates per treatment. Leaf color, leaf area, plant quality, K^+^/Na^+^ ratio, TNC, RSC, and proline content were analyzed to evaluate differences among treatments during the recovery period. When significant differences were detected, treatment means were separated using the least significant difference (LSD) test at the 0.05 probability level.

## 5. Conclusions

Exogenous application of growth regulators significantly enhanced post-salinity recovery in *Arum palaestinum* Boiss., with clear differences in efficacy among treatments. Abscisic acid (2.0 mM) was the most effective regulator, followed by ethephon (20.0 mM), while salicylic acid (2.0 mM) and fusicoccin (0.03 mM) exerted moderate but beneficial effects. ABA-treated plants exhibited superior recovery, demonstrated by improved leaf color, leaf area, chlorophyll content, overall plant quality, ionic homeostasis, and carbohydrate accumulation, accompanied by marked reductions in stress-related metabolites. These responses underscore the central role of ABA in orchestrating physiological and metabolic reprogramming during recovery from salinity stress, including enhanced ion regulation, maintenance of photosynthetic efficiency, and optimized carbon allocation, while ethylene contributed to recovery primarily through modulation of osmotic balance and stress signaling. The observed hierarchy in treatment effectiveness highlights the potential of hormone-based interventions for mitigating salinity damage and restoring plant growth. Overall, these findings provide a robust physiological foundation for the targeted use of ABA- and ethylene-based treatments to improve salinity tolerance and post-stress performance in *A. palaestinum* and offer a valuable framework for developing hormone-mediated strategies in other salt-sensitive medicinal and ornamental species.

## Figures and Tables

**Table 1:** Analysis of variance (mean squares) for leaf color, leaf area, plant quality, relative chlorophyll content (SPAD), shoot K^+^/Na^+^ ratio, total nonstructural carbohydrates (TNC), reducing sugars content (RSC), and proline content of *Arum palaestinum* during recovery from salinity stress.

Parameter	Growth Regulators (G)	Growth Regulator Concentrations (C)	G × C
Leaf color	7.5 **	90.2 **	50.2 *
Leaf area	11.5 **	402 **	44.6 *
Plant quality	6.9 **	514 **	54.2 *
Chla	36.0 **	574.0 **	303.0 *
K^+^/Na^+^	1.6 **	24.0 **	25.0 *
TNC	912.0 **	1005.0 **	1310.0 *
RSC.	34.0 **	370.0 **	215.0 *
Proline	1040.0 **	1370.0 **	1330.0 *

* Significant at *p* ≤ 0.05; ** significant at *p* ≤ 0.01.

## Data Availability

The original contributions presented in this study are included in the article. Further inquiries can be directed to the corresponding author.

## Abbreviations

The following abbreviations are used in this manuscript:

PGRs	Plant Growth Regulators
FC	Fusicoccin
ABA	Abscisic Acid
SA	Salicylic Acid
Chl	Chlorophyll
SOS	Salt Overly Sensitive
RSC	Reducing Sugars Content
TNC	Total Nonstructural Carbohydrates
